# Ethnic differences in timely adjuvant chemotherapy and radiation therapy for breast cancer in New Zealand: a cohort study

**DOI:** 10.1186/1471-2407-14-839

**Published:** 2014-11-18

**Authors:** Sanjeewa Seneviratne, Ian Campbell, Nina Scott, Marion Kuper-Hommel, Glenys Round, Ross Lawrenson

**Affiliations:** Waikato Clinical School, University of Auckland, Breast Cancer Research Office, Waikato Hospital, PO Box 934, Hamilton, 3240 New Zealand; Department of Surgery, University of Colombo, Colombo, Sri Lanka; Māori Health Services, Waikato District Health Board, Hamilton, New Zealand; Department of Oncology, Waikato District Health Board, Hamilton, New Zealand

**Keywords:** Breast cancer, Chemotherapy, Radiation therapy, Delay, Ethnicity, Inequity

## Abstract

**Background:**

Indigenous and/or minority ethnic women are known to experience longer delays for treatment of breast cancer, which has been shown to contribute to ethnic inequities in breast cancer mortality. We examined factors associated with delay in adjuvant chemotherapy and radiotherapy for breast cancer, and its impact on the mortality inequity between Indigenous Māori and European women in New Zealand.

**Methods:**

All women with newly diagnosed invasive non-metastatic breast cancer diagnosed during 1999–2012, who underwent adjuvant chemotherapy (n = 922) or radiation therapy (n = 996) as first adjuvant therapy after surgery were identified from the Waikato breast cancer register. Factors associated with delay in adjuvant chemotherapy (60-day threshold) and radiation therapy (90-day threshold) were analysed in univariate and multivariate models. Association between delay in adjuvant therapy and breast cancer mortality were explored in Cox regression models.

**Results:**

Overall, 32.4% and 32.3% women experienced delays longer than thresholds for chemotherapy and radiotherapy, respectively. Higher proportions of Māori compared with NZ European women experienced delays longer than thresholds for adjuvant radiation therapy (39.8% vs. 30.6%, p = 0.045) and chemotherapy (37.3% vs. 30.5%, p = 0.103). Rural compared with urban residency, requiring a surgical re-excision and treatment in public compared with private hospitals were associated with significantly longer delays (p < 0.05) for adjuvant therapy in the multivariate model. Breast cancer mortality was significantly higher for women with a delay in initiating first adjuvant therapy (hazard ratio [HR] =1.45, 95% confidence interval [CI] 1.05-2.01). Mortality risks were higher for women with delays in chemotherapy (HR = 1.34, 95% CI 0.89-2.01) or radiation therapy (HR = 1.28, 95% CI 0.68-2.40), although these were statistically non-significant.

**Conclusions:**

Indigenous Māori women appeared to experience longer delays for adjuvant breast cancer treatment, which may be contributing towards higher breast cancer mortality in Māori compared with NZ European women. Measures to reduce delay in adjuvant therapy may reduce ethnic inequities and improve breast cancer outcomes for all women with breast cancer in New Zealand.

## Background

Ethnic disparities in receipt of breast cancer care are well documented, and have been shown to contribute towards worse breast cancer outcomes among Indigenous and/or minority ethnic women [1, 2]. Indigenous and/or minority ethnic women are more likely to experience longer delays in initiation of treatment for breast cancer [3–5], which are known to increase risks of breast cancer recurrence and mortality [6–9].

Indigenous Māori, who make up about 15% of New Zealand population, carry a greater burden of breast cancer due to a higher incidence and a lower survival compared with NZ European women [10]. Advanced cancer stage at diagnosis in Māori women is known to have the greatest impact on breast cancer survival disparity between these two groups. Still, stage adjusted breast cancer mortality is about 30% higher for Māori women indicating a significant contribution from factors other than stage at diagnosis, including possible differences in treatment quality and delays in treatment [10].

A substantial reduction in breast cancer mortality has been observed in developed countries over the last two decades, which has been attributed to earlier diagnosis with widespread use of screening mammography and advances in breast cancer treatment [11]. Timeliness of instituting treatment is crucial in order to obtain the maximum potential benefit from these new and advanced treatments. Two recent meta-analyses have shown a 6% and 15% increase in relative mortality rate with each 4-week delay in initiating adjuvant chemotherapy [12, 13]. Although timeline thresholds given in treatment guidelines are sometimes arbitrary and controversial, longer delays for surgery, chemotherapy and radiation therapy have all been proven to be associated with poorer breast cancer outcomes including higher risks of recurrence and mortality [6, 8, 9, 12, 13].

In New Zealand, longer delays experienced by Māori in the receipt of cancer care have been reported for surgical treatment of breast and lung cancer [14, 15] and for receipt of adjuvant chemotherapy for bowel cancer [16]. To date, no data are available on delays in adjuvant therapy experienced by New Zealand women with breast cancer or ethnic differences in the receipt of such treatment.

We conducted this study to identify ethnic differences in delay in initiating adjuvant chemotherapy or radiation therapy following surgical treatment for invasive breast cancer. We also explored time trends in delays and impact of delay on breast cancer outcomes in this cohort of women with breast cancer.

## Methods

### Study population

Data for this study were extracted from the Waikato breast cancer register (WBCR), a population based, prospective, comprehensive database of newly diagnosed breast cancers in the Waikato, New Zealand since 1999. The WBCR includes over 98% of all diagnosed cancers in the region and validity of its data has been reported previously [17]. All newly diagnosed invasive female breast cancers during the period from 01/01/1999 through 31/12/2012, were identified from the WBCR (n = 2848). Of this, women with metastatic cancer (stage IV disease) at diagnosis (n = 166), women who did not undergo primary surgery (n = 114) and women who received neo-adjuvant therapy (n = 87), were excluded.

### Healthcare system in New Zealand and breast cancer services in the Waikato

New Zealand has a well-resourced publicly funded national health system that provides specialist and hospital care to all citizens without patient charges. Parallel to the public system, there are a variety of private hospital facilities available, which are mostly funded through insurance schemes. A national breast cancer screening programme, BreastScreen Aotearoa (BSA) provides free biannual breast cancer screening for all women aged 45–69 years. The Waikato region with a population of 365,000 is the fourth largest of twenty District Health Boards in New Zealand. It has a major urban centre, a significant rural population and a Māori population of almost 80,000. Public sector breast cancer services in the Waikato are provided through specialist services at the district tertiary hospital in Hamilton. In addition, surgical treatment is also provided through several well-equipped private hospitals. Radiation therapy services for the Waikato region are provided exclusively through the radiation facility at the tertiary hospital in Hamilton, while chemotherapy facilities are provided through a satellite site in addition to the tertiary hospital in Hamilton.

### Data

Patient ethnicity was obtained from the WBCR, which records self-assigned ethnicity as declared by each patient during the WBCR consent process. Ethnicity was grouped into four categories; Māori, Pacific (including Samoan, Cook Island Māori, Tongan, Niuean, Tokelauan, Fijian and other Pacific Islands), NZ European, and ‘Other’. Cancer staging was performed according to TNM (Tumour, Lymph node and Metastasis) staging system [18].

Socioeconomic status of each woman was categorized according to the New Zealand Deprivation Index 2006 (NZDep06) [19]. NZDep06 measures socioeconomic status based on area of residence and assigns a deprivation score on a scale from 1 to 10 (1-least deprived 10% of areas, 10-most deprived 10% of areas in New Zealand) based on nine parameters measured during the population census in 2006. Distance from a woman’s residence to treatment facility where surgery was carried out was calculated based on New Zealand Statistics [20] and was categorized into four categories; 0-10 km, 10-50 km, 50–100 and >100 km.

A comorbidity score for each woman was calculated using Charlson Comorbidity Index [21], based on existing comorbidities at the time of diagnosis of breast cancer. Comorbidity score was categorized in to 0 or ≥1.

### Delay in adjuvant therapy

To assess time gap from surgery to initiation of first adjuvant therapy (i.e. chemotherapy or radiation therapy), all women with non-metastatic invasive breast cancer undergoing surgery as primary breast cancer treatment modality were identified (n=2481). Chemotherapy was considered as the first adjuvant therapy for all eligible women undergoing adjuvant chemotherapy (n=922, 37.2%) and radiation therapy was considered as the first adjuvant therapy for women undergoing radiation therapy without prior adjuvant chemotherapy (n=996, 40.1%). The time gap to adjuvant chemotherapy and radiation therapy was defined as number of days from the most definitive operation for the breast cancer to the first administration of chemotherapy or radiation therapy [5]. The definitive surgical procedure at the primary site captured the most invasive surgical procedure at the primary site and included excisional biopsy, wide local excision and mastectomy. Women who had delays of more than 365 days for either chemotherapy or radiation therapy were excluded.

A threshold of 60 days was used as the acceptable threshold delay for initiating chemotherapy, based on evidence from three recently published papers. These include two meta-analyses which have demonstrated 6% and 15% worse overall and disease free relative mortality rates for each 4-week delay in initiating chemotherapy [12, 13]. A third study from the USA, which included more than 6000 women found significantly worse disease free survival for women with stage II-III or triple negative or HER-2 positive cancers, who experienced delays longer than 60 days [9]. As some previous studies have used a 90-day threshold delay for chemotherapy [7, 22–24], we performed additional analyses with a 90-day threshold for chemotherapy. For radiation therapy, a 90-day threshold was used, which has conventionally been used in the assessment of radiation therapy delay [8].

### Data analysis

Data were analysed using SPSS (version 22). Continuous variables were summarized as mean/median with standard deviation (SD). Independent samples median test was used to test differences in continuous variables. Chi squared tests (χ^2^) for trend was used to test differences in delay among groups including age, ethnicity, stage, mode of diagnosis (screen detected or symptomatic) and year of diagnosis. Multivariable logistic regression analyses were performed to estimate independent association between above factors and delays in initiating adjuvant therapy. Separate Cox regression models were used to identify the association between breast cancer specific mortality and delay (overall, chemotherapy and radiation therapy) adjusting for covariates.

Ethical approval for this study was obtained from the New Zealand Northern ‘A’ Ethics Committee (Ref. No. 12/NTA/42).

## Results

This study included a total of 1918 women of whom 922 (711 NZ European and 153 Māori) received chemotherapy and 996 (853 NZ European and 113 Māori) received radiation therapy as first adjuvant therapy. The median time gap for initiating adjuvant chemotherapy was 49 days (mean 52.6, SD 21.3) and for adjuvant radiation therapy was 76 days (mean 81.4, SD 32.5). Māori women experienced significantly longer median delays compared with NZ European women for both adjuvant chemotherapy (median delay 54 vs. 49 days, p = 0.017) and radiation therapy (median delay 83 vs. 75 days, p = 0.046). Overall, 318 (31.9%) women experienced a delay longer than 90 days to receive radiation therapy and the number of women who did not receive chemotherapy within 60-day threshold was 301 (32.4%). A total of 619 (32.3%) women experienced a delay in receiving first adjuvant therapy. Five percent (n = 46) women experienced a delay longer than 90 days for chemotherapy. A significantly higher proportion of Māori women experienced a delay longer than 90 days compared with NZ European women (8.7% vs. 4.2%, p = 0.025).

Univariate analysis of factors associated with delay in receiving first adjuvant therapy, chemotherapy and radiation therapy are shown in Table 1 and the multivariable logistic regression in Table 2. Māori or Pacific ethnicity compared with NZ European ethnicity, earlier year of diagnosis, requiring a re-excision following primary surgery, longer distance from the tertiary care hospital and receiving surgical treatment from a public versus private hospital were associated with significantly longer delays (p < 0.05) for first adjuvant therapy in both unadjusted and adjusted models. For chemotherapy, a significant inverse association (p = 0.048) was observed between stage and proportion with delays longer than 60 days with the smallest proportion observed for stage III disease. Delays longer than threshold limits for chemotherapy and radiation therapy were significantly associated with re-excisions after primary surgery and treatment in public hospital in both univariate and multivariate models. Distance from treatment facility was significantly associated with delay in radiation therapy (p = 0.021), but not for delay in chemotherapy (p = 0.540). Delay for radiation therapy has significantly reduced over time (p < 0.001), while delays for chemotherapy have increased during 1999–2009, although a decline is observed over 2010–2012.

**Table 1:** **Factors associated with delay in adjuvant therapy**

Characteristic	Total (N = 1918)	Delay in first adjuvant therapy ^a^		Delay in radiation therapy >90 days		Delay in chemotherapy >60 days	
	n (%)	n (%)	p	n (%)	p	n (%)	p
Ethnicity							
NZ European	1564 (81.5)	478 (30.6)		261 (30.6)		217 (30.5)	
Māori	266 (13.9)	101 (38.0)	0.022	45 (39.8)	0.045	57 (37.3)	0.103
Pacific	38 (2.0)	19 (50.0)	0.013	6 (54.5)	0.101	13 (48.1)	0.057
Other	50 (2.6)	21 (42.0)	0.112	7 (35.0)	0.676	14 (46.7)	0.065
Age (years)			0.846		0.704		0.091
<40	117 (6.1)	38 (32.5)		9 (40.9)		29 (30.5)	
40-49	433 (22.6)	130 (30.0)		43 (32.8)		87 (28.8)	
50-59	583 (30.4)	193 (33.1)		89 (34.2)		104 (32.2)	
60-69	494 (25.8)	165 (33.4)		94 (29.2)		71 (41.3)	
70-79	212 (11.1)	70 (33.0)		60 (33.0)		10 (33.3)	
80+	79 (4.1)	23 (29.1)		23 (29.1)		0	
Stage at diagnosis			<0.001		<0.001		0.048
I	800 (41.7)	228 (28.5)		176 (27.0)		52 (35.4)	
II	772 (40.3)	291 (37.7)		112 (43.1)		179 (35.0)	
III	346 (18.0)	100 (28.9)		30 (36.1)		70 (26.6)	
Year of diagnosis			<0.001		<0.001		<0.001
1999-2002	393 (20.5)	166 (42.2)		105 (62.5)		61 (27.1)	
2003-2006	579 (30.2)	181 (31.3)		107 (35.9)		74 (26.3)	
2007-2009	452 (23.6)	146 (32.3)		50 (20.0)		96 (47.5)	
2010-2012	494 (25.8)	126 (25.5)		56 (20.0)		70 (32.7)	
Screening status			0.162		0.005		0.247
Non-screen	1110 (57.9)	372 (33.5)		171 (36.3)		201 (31.5)	
Screen detected	808 (42.1)	247 (30.6)		147(28.0)		100 (35.3)	
Deprivation			0.257		0.795		0.147
Dep 1-2	213 (11.1)	55 (25.8)		29 (28.7)		26 (23.2)	
Dep 3-4	206 (10.7)	66 (32.0)		36 (34.3)		30 (29.7)	
Dep 5-6	487 (25.4)	161 (33.1)		75 (29.8)		86 (36.6)	
Dep 7-8	532 (27.7)	174 (32.7)		98 (32.7)		76 (32.8)	
Dep 9-10	480 (25.0)	163 (34.0)		80 (33.6)		83 (34.3)	
Primary surgery			0.913		0.014		0.058
BCS	1318 (68.7)	425 (32.2)		260 (30.4)		165 (35.6)	
Mastectomy	600 (31.3)	194 (32.3)		58 (40.8)		136 (29.7)	
Re-excision			<0.001		<0.001		0.002
No	1659 (86.5)	491 (29.6)		250 (28.6)		241 (30.7)	
Yes	259 (13.5)	128 (49.4)		68 (55.3)		60(44.1)	
Distance from hospital			0.019		0.021		0.540
<10 km	630 (32.8)	192 (30.5)		94 (28.1)		98 (33.1)	
10-50 km	740 (38.6)	221 (29.9)		107 (29.2)		114 (30.5)	
50-100 km	462 (24.1)	169 (36.6)		100 (38.9)		69 (33.7)	
>100 km	86 (4.5)	37 (43.0)		17 (43.6)		20 (42.6)	
Surgical facility type			<0.001		0.009		0.001
Private	632 (33.0)	163 (25.8)		73 (25.8)		90 (25.8)	
Public	1286 (67.0)	456 (35.5)		245 (34.4)		211 (36.8)	
Charlson score			0.175		0.434		0.520
0	1677 (87.4)	534 (31.8)		265 (31.7)		269 (32.0)	
1+	241 (12.6)	85 (35.3)		53 (33.3)		32 (39.0)	

(Univariate analysis for factors associated with delay in first adjuvant therapy ^a^, delay in radiation therapy longer than 90 days and delay in chemotherapy longer than 60 days for women with newly diagnosed invasive breast cancer in the Waikato, New Zealand during 1999-2012.

^a^delay in radiation therapy longer than 90 days or delay in chemotherapy longer than 60 days.

**Table 2:** **Multivariable model for factors associated with delay in adjuvant therapy**

		Delay in first adjuvant therapy ^a^			Delay in radiation therapy >90 days			Delay in chemotherapy >60 days		
		OR	95% CI	p	OR	95% CI	p	OR	95% CI	p
Ethnicity										
NZ European		Ref.			Ref			Ref		
Māori		1.32	0.98-1.77	0.069	1.87	1.17-3.02	0.010	1.17	0.78-1.74	0.452
Pacific		2.05	1.03-4.08	0.041	2.47	0.54-11.2	0.242	1.87	0.82-4.26	0.136
Other		1.71	0.94-3.11	0.082	1.32	0.46-4.05	0.564	2.03	0.93-4.44	0.076
Year of diagnosis^b^		0.79	0.72-0.87	<0.001	0.49	0.42-0.56	<0.001	1.17	1.03-1.34	0.017
Re-excision		2.47	1.85-3.28	<0.001	3.55	2.32-5.44	<0.001	1.81	1.19-2.73	0.005
Surgical facility type^c^		1.53	1.22-1.93	<0.001	1.59	1.12-2.26	0.010	1.58	1.15-2.16	0.005
Distance from hospital^d^		1.10	1.02-1.18	0.024	1.23	1.09-1.39	0.001	1.03	0.92-1.15	0.654

(Multivariable logistic regression model for factors associated with delay in first adjuvant therapy, delay in radiation therapy longer than 90 days and delay in chemotherapy longer than 60 days adjusting for age, tumour stage, socioeconomic deprivation and comorbidity score).

^a^Delay in radiation therapy longer than 90 days or delay in chemotherapy longer than 60 days, ^b^Year categories as in Table 1 and reference category year 1999–2002, ^c^Reference category private surgical facility, ^d^Reference category <10 km.

Adjusted multivariable logistic regression model identified year of diagnosis, re-excision and surgical treatment facility type to be independently associated with delay in first adjuvant therapy as well as for delay in chemotherapy and radiation therapy (Table 2). Overall, Māori, Pacific and Other ethnicity were associated with higher likelihoods of delay for chemotherapy, radiation therapy and for first adjuvant therapy, although this was statistically significant only for delay in radiotherapy for Māori and delay in first adjuvant therapy for Pacific women in the multivariable model. Sensitivity analysis with 90-day chemotherapy delay threshold yielded similar results in the multivariable regression model (data not shown) with year of diagnosis (OR = 1.37, p < 0.001), re-excision (OR = 3.96, p = 0.001) and public hospital care (OR = 4.89, p = 0.001) showing significant associations. Māori women had a non-significantly higher risk for a chemotherapy delay longer than 90 days (OR = 1.41, p = 0.291) compared with NZ European women in this model.

**Figure 1:**
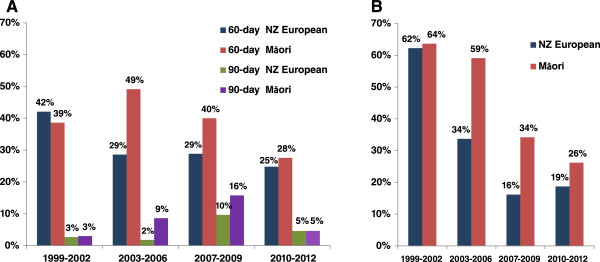
**Time trends in delay in adjuvant therapy (time trends in delay in adjuvant chemotherapy longer than 60 and 90 days (Panel A) and adjuvant radiation therapy longer than 90 days (Panel B) for invasive breast cancer in the Waikato, New Zealand 1999–2012).**

Time trends in 60-day chemotherapy and 90-day radiation therapy delay by ethnicity is shown in Figure 1. Higher proportions of Māori women have consistently experienced longer delays for radiation therapy compared with NZ European women over the study period which were significant during 2003–2006 and 2007–2009 periods (p = 0.010 and p = 0.012, respectively). The reduction in radiation therapy delay has been greater for NZ European than for Māori over 1999–2009, which has resulted in a widening of disparity in delay between Māori and NZ European, although this gap seems to have narrowed over the last three year period of the study. Higher proportions of Māori have experienced delays longer than 60 days for chemotherapy over 1999–2009 period, but since has declined below the rate for NZ European women over 2010–2012. For delays in chemotherapy longer than 90 days (Figure 1), the highest proportion was seen during 2007–2009 period (overall 10.9%, NZ European 9.7%, Māori 15.8%) and since has declined to 5.2% (NZ European 4.6%, Māori 6.8%) during 2010–2012.

A survival analysis using a multivariable Cox regression analysis adjusting for covariates showed a significantly higher breast cancer specific mortality risk (HR = 1.45, p = 0.024) among women who experienced delays in first adjuvant therapy (Table 3). A sensitivity analysis with a 90-day delay threshold for chemotherapy yielded a similar increased trend for breast cancer mortality for women who experienced a delay for first adjuvant therapy (HR = 1.29, 0.81-2.05, p = 0.288), although this difference was no longer statistically significant. Delay in chemotherapy (HR = 1.34, p = 0.157) and radiation therapy (HR = 1.28, p = 0.449) were also tended to be associated with higher hazards of breast cancer mortality although these did not reach statistical significance.

**Table 3:** **Proportional hazard models for breast cancer specific mortality**

	HR ^ab^	95% CI	p
Delay in first adjuvant therapy	1.45	1.05-2.01	0.024
Delay in radiotherapy >90 days	1.28	0.68-2.40	0.449
Delay in chemotherapy >60 days	1.34	0.89-2.01	0.157

(Cox proportional models for breast cancer specific mortality by delay in first adjuvant therapy, radiation therapy and chemotherapy).

^a^Performed in three separate Cox regression models, ^b^For stage I-III invasive breast cancer adjusted for age, ethnicity, stage of disease (i.e. tumour size and number of positive lymph nodes), tumour grade, oestrogen receptor status, lympho-vascular invasion, year of diagnosis, comorbidity score and receipt of adjuvant therapy.

## Discussion

From this study we found that among women with non-metastatic invasive breast cancer in the Waikato, New Zealand, almost a third (32.3%) experienced a delay in initiating radiation therapy or chemotherapy as first adjuvant therapy following primary surgical treatment. Furthermore, Māori and Pacific compared with NZ European, rural compared with urban dwelling women and women who received surgical treatment in public compared with private hospitals had significantly higher likelihoods of experiencing delays longer than thresholds for adjuvant therapy. Increasing socioeconomic deprivation tended to be non-significantly associated with longer delays in adjuvant therapy while no association was observed between delay and patient age. Although delay in radiation therapy seems to have improved over time, substantial proportions of women continue to experience clinically significant delays for both chemotherapy and radiation therapy.

Delays in adjuvant therapy for breast cancer experienced by disadvantaged populations including minority and Indigenous ethnic groups are well documented [1, 2]. From a study based on over 100,000 women from the US National Cancer Database, Fedewa and colleagues reported that Black African women were 30% and 50% more likely to experience delays longer than 60 and 90 days respectively, for initiation of adjuvant chemotherapy compared with White European women [5]. We observed a similar pattern where greater proportions of Māori women experienced longer delays for chemotherapy (23% higher for 60-day and 100% higher for 90-day delay) compared with NZ European women. However, from 2007–2009, there were no significant inequities between Māori and NZ European women and, in 2010–2012 Māori women were less likely to experience a delay in accessing chemotherapy. Timeliness in initiating treatment for breast cancer is of greater importance for women with more advanced or more aggressive cancers (i.e. stage II or III, hormone receptor negative, HER-2 positive) [9, 25]. Māori women are more likely to be diagnosed with more advanced disease and are more likely to have hormone receptor negative and HER-2 positive breast cancers [10, 26], and hence longer delays in adjuvant therapy, as demonstrated in this study are likely to have a greater impact on breast cancer survival in Māori women. However, women with more advanced cancers seem to have had shorter delays, possibly due to prioritized care for these higher risk women and, hence likely to have had a minimal differential impact on higher mortality in Māori compared with NZ European women.

Delays in cancer adjuvant therapy are associated with factors including lack of access to healthcare, difficulties with navigating the health system, geographic distance to treatment facility, availability of transport and ability to take time-off work to attend adjuvant therapy [1, 27, 28]. Further, women of some ethnic minority populations including Black Africans in the USA have been shown to be less willing to undergo adjuvant treatments because of greater fear of side-effects and lack of knowledge on potential benefits [29, 30]. Longer delays for adjuvant therapy observed among Māori compared with NZ European women in this study were likely due to a combination of these factors as Māori are more likely to be socioeconomically deprived and live in rural areas with less access to transport compared with NZ Europeans [31]. These differences were observed despite temporary accommodation and/or free transport been provided by the Waikato District Health Board for women requiring these facilities to attend adjuvant chemotherapy and radiation therapy. Furthermore, the higher risk of Māori compared with NZ European women for longer delays persisted even after adjusting for deprivation and residence which probably was due to the impact of unmeasured or under-measured confounders in the present study. For instance, Māori women are more likely to have lower levels of education, lower health literacy and are less likely to have a health insurance policy compared with NZ European women [31], factors which are known to be associated with longer delays in cancer treatment [5]. These factors were not included in our analyses due to unavailability of these data from the WBCR. We also observed that significantly smaller proportions of women who received surgical treatment in the private sector had experienced delays beyond the threshold limits for both chemotherapy and radiation therapy compared with women treated in the public sector. Ability to afford treatment from private sector has a strong correlation with higher socioeconomic status and/or having a health insurance policy. Hence, this observation supports, though indirectly, affluent socioeconomic background and health insurance as factors influencing shorter delays in the receipt of adjuvant therapy. This disparity is observed, despite more than 95% women included in the present study receiving their adjuvant chemotherapy and radiation therapy free of charge from the public sector.

Rural residency is known to influence delay as well as a woman’s decision to undergo adjuvant radiation therapy for breast cancer [32]. Radiation therapy requires women to attend a radiation facility five days a week over a period ranging from three to six consecutive weeks and due to this many rural women prefer mastectomy over BCS [14, 32]. This is of greater importance for the study women as the Waikato district health board covers an area of over 20,000 square kilometres and yet has provided radiation therapy services through a single central facility. In comparison, chemotherapy in most instances requires only once in three weeks and/or once a week visits to a chemotherapy facility and is less likely to be influenced by rural residence. Consistent with this, no significant differences in delays for chemotherapy were observed between urban and rural women in the present study.

Several effective strategies for minimizing delays in adjuvant therapy and reducing inequities in delay between socioeconomic and ethnic groups are reported in literature. These include improving access through increasing supply or efficient usage of existing cancer resources through coordinated cancer care, decentralization of cancer services and through improving patient health literacy [33–36]. As we have observed, almost a third of women have experienced delays in adjuvant therapy beyond the threshold limits and it appears that overloading of oncology services was a likely factor. The greatest proportion of women experiencing delays longer than three months for radiation therapy was seen during 1999–2002 which was a time when a severe nationwide shortage of radiation therapy services was experienced in New Zealand [37]. Since then the supply of radiation facilities has increased resulting in a gradual reduction in proportion of women experiencing delays longer than three months. However, the increase in supply of radiation therapy facilities has been inadequate or lagging behind to keep up with the increase in number of patients requiring radiation therapy. As a result, even in 2010–2012, about 20% women were observed to have experienced delays longer than three months for radiation therapy. Delays in chemotherapy appeared to have worsened during 1999–2009 followed by an improvement in the last time period. These different patterns of delay in chemotherapy and radiation therapy may reflect issues at national level as well as local issues of service capacity compared with demand.

Increasing the supply of oncology services alone are unlikely to eliminate inequities in delay, as disadvantaged women (i.e. ethnic minority, socioeconomically deprived, rural, etc.) will still be more likely to be subjected to longer delays. Improved patient navigation through cancer care coordinators (CCC) has been shown to help reduce delays, especially for women who are at-risk for longer delays which include women of minority ethnicity and low socioeconomic groups [38]. The Waikato District Health Board has two full-time CCC’s providing support for women with breast cancer since 2009. It is likely that CCCs have made a major contribution towards the observed reduction in delay overall and reduction in inequities between Māori and NZ European women observed during 2010–2012. The Ministry of Health has also identified CCC’s as a key strategy to increase quality and reduce inequalities in cancer care, and since 2012 has provided funding for all District Health Boards to employ CCC’s for management of common cancers in New Zealand [39].

This study did not examine the type, duration, dose or rates of completion of adjuvant chemotherapy or radiation therapy which are also known to impact on the efficacy of these adjuvant therapies [40, 41]. Further, although we have observed several associations with longer delays, we were unable to identify causes for these delays i.e. whether delays were due to longer wait time for appointments or due to patients not attending appointments, additional investigations required due to patient comorbidity, etc. Another limitation of this study was the inclusion of only small numbers of Pacific and Other women. Although we have observed longer delays among Pacific women small sample size prevented further analyses. Further, we have used an area based deprivation system as a proxy measure for individual socioeconomic deprivation. Although the NZDep2006 has been validated as a proxy measure for individual derivation [42], it inherently has a limited ability to predict individual deprivation. Lack of a significant correlation between deprivation score and delay in adjuvant therapy as observed in this study could have been influenced by this lack of precision. In addition, although the WBCR data are highly complete [17], minor errors in misclassifications or data entry are inevitable. A stringent quality control process including a regular audit of the database is expected to have minimized, but not eliminated such errors.

## Conclusions

In conclusion, we have observed significantly longer delays experienced by Indigenous Māori women, rural women and women receiving surgical care in the public sector. Although delays in adjuvant therapy appear to have improved over the study period, it is concerning to note the substantial proportion of women continuing to experience clinically significant delays for adjuvant breast cancer therapy. Reducing delays through improvements in availability, efficiency and access to oncology services will not only minimize ethnic inequities in delay but may improve outcomes for all women with breast cancer in New Zealand.
